# Lavender (*Lavandula angustifolia*) syrup as an adjunct to standard care in patients with mild to moderate COVID-19: An open-label, randomized, controlled clinical trial 

**DOI:** 10.22038/AJP.2022.21606

**Published:** 2023

**Authors:** Marzieh Qaraaty, Mohsen Bahrami, Sadegh-Ali Azimi, Fataneh Hashem-Dabaghian, Safoora Saberi, Syed Mohd Abbas Zaidi, Amirhossein Sahebkar, Ayesheh Enayati

**Affiliations:** 1 *Clinical Research Development Unit (CRDU), Sayad Shirazi Hospital, Department of Persian Medicine, School of Medicine, Golestan University of Medical Sciences, Gorgan, Iran*; 2 *Department of Persian Medicine, School of Medicine, Golestan University of Medical Sciences, Gorgan, Iran*; 3 *Academic Center for Education Culture and Research, Tehran, Iran *; 4 *Infectious Diseases Research Center, Golestan University of Medical Sciences, Gorgan, Iran*; 5 *Department of Traditional Medicine, Institute for Studies in Medical History, Persian and Complementary Medicine, School of Persian Medicine, Iran University of Medical Sciences, Tehran, Iran *; 6 *Gonbad-E-Kavoos Health Center, Golestan University of Medical Sciences, Gonbad-E-Kavoos, Iran*; 7 * H.S.Z.H. Government Unani Medical College, Bhopal (M.P), India *; 8 *Applied Biomedical Research Center, Mashhad University of Medical Sciences, Mashhad, Iran*; 9 *Biotechnology Research Center, Pharmaceutical Technology Institute, Mashhad University of Medical Sciences, Mashhad, Iran*; 10 *Ischemic Disorders Research Center, Golestan University of Medical Sciences, Gorgan, Iran*

**Keywords:** COVID-19, Cough, Herbal medicine, Lavandula angustifolia, Persian medicine

## Abstract

**Objective:**

ongoing COVID-19 pandemic has been associated with clinical signs characterized by fever, fatigue and cough. Our study aimed to assess the efficacy of a Persian medicine formulation, lavender syrup, as an add-on to standard care in patients with mild to moderate COVID-19.

**Materials and Methods:**

In this clinical trial which was conducted in Gorgan (Iran), 84 male and female COVID-19 outpatients were randomly allocated to either lavender syrup receiving 9 ml/twice/day for 21 days with standard conventional care or control groups. The primary objectives were to assess the improvement of clinical symptoms, while the secondary objectives were treatment satisfaction and anxiety levels which were evaluated once a week for 3 weeks.

**Results:**

Out of 84 participants, 81 were analyzed (41 in the add-on group). The comparison between groups for cough severity and anosmia showed a higher reduction in the lavender group. The effect size was 0.6 for cough relief. Other symptoms and the Hamilton total score decreased in both groups with no statistically significant differences between the groups. The lavender group showed greater patients’ satisfaction score.

**Conclusion:**

Adjunctive therapy with lavender syrup could reduce cough and improve the quality of life in patients with COVID-19 patients.

## Introduction

The COVID-19 pandemic led to a global health problem at the beginning of 2020 (Kannan et al., 2020; Shah et al., 2020; Tabata et al., 2020; Xia et al., 2021). COVID-19 initially and mainly transmits through the upper respiratory tract, which induces severe acute respiratory syndrome. Additionally, previous reports of COVID-19 introduced fever, cough, and dyspnea, as the most common clinical symptoms in people who were infected with COVID-19 (Kannan et al., 2020; Shah et al., 2020; Xia et al., 2021). Because there is a massive increase in confirmed or highly-suspicious COVID-19 cases, it is crucial to adopt an adjuvant approach with the aim of improving the disease prognosis and supporting the already overstretched health care system for the management of COVID-19.

Herbal medicines are widely used in many traditional systems for the management of various diseases (Yang, 2020). These herbal medicines might be of great importance, especially when the currently used anti-viral and other supporting drugs do not offer any significant relief to COVID-19 patients (Cao et al., 2020; Ferner and Aronson, 2020) and hence, the use of potential active traditional herbal medicines might be a promising candidate that should be tested at least to prevent and control COVID-19 symptoms in mild-to-moderate COVID-19 patients (Yang, 2020). 

Three herbal patents and clinical trials were approved that traditional herbal medicine attenuated the most common COVID-19 symptoms, including fever, cough, and fatigue, and reduced severe conditions of patients (Karimi et al., 2021; Tavakoli et al., 2022).

Persian medicine which is popularly known as Unani medicine in India, Sri Lanka, Bangladesh, and some other countries, is an ancient system of medicine, which mainly uses plant-based medicines. Among these plant-based ancient formulations, some studies have reported the beneficial effects of “*Ostukhudus”* (*Lavandula angustifolia* L.) of the family Lamiaceae on cold, asthma, bronchitis, chest pain, shortness of breath and cough, along with its antioxidant and anti-inflammatory properties (Almohawes and Alruhaimi, 2019; Anushiravani et al., 2018; Ezzoubi et al., 2014; Kähler et al., 2019; Khorasani, 2012). In addition, a double-blind randomized clinical trial on adult patients with acute bronchitis showed that oral administration of lavender essential oil capsule (2 capsules/3 times/day, for 10 days) can ameliorate the bronchitis severity score compared with the placebo group and improve additional signs (including cough, sputum production, rales/rhonchi, chest pain during coughing, dyspnea, and patient’s satisfaction) (Kähler et al., 2019). 

Because of several years history of use of this medicinal herb and its safety and efficacy (Kähler et al., 2019), we designed an open-labeled, randomized controlled study to investigate the effect of lavender (*L. angustifolia*) syrup combined with conventional medicine on improving the severity of cough and clinical symptoms in patients with COVID-19.

## Materials and Methods

**Study design**

We conducted an open-label randomized controlled trial on COVID-19 patients from July 2020 to September 2020. Patients with COVID-19 who were admitted to the Gonbad-E-Kavoos Health center, Golestan University of Medical Sciences, Iran, participated in the trial.

**Study population**

Highly probable patients of COVID-19 between 18 and 65 years of age having fever≥38℃, oxygen saturation, or a feeling of hot flashes or one of the main clinical symptoms of dry cough, headache, malaise, weakness, lethargy, anosmia (olfactory disorder) or taste disturbance, anorexia, and candidate for outpatient treatment entered into the trial. Exclusion criteria were having respiratory distress, pregnancy or lactation, smoking, use of other herbal medicines to control the symptoms of the disease, sensitivity to lavender, or need for hospitalization.

**Ethics**

The trial has been approved by the Ethics Committee of Golestan University of Medical Sciences (IR.GOUMS.REC.1399.025) and registered in Iranian Registry for Clinical Trials (IRCT20110907007511N4). All patients signed the written informed consent to participate in the trial. 

**Formulation of the syrup**

**Lavender syrup preparation**

Based on “Makhzan al-Advieh” (the Storehouse of Medicaments), a Persian medicine book, it was found that *L. angustifolia* has been recommended for symptoms that resemble those of COVID-19 (Aghili, 2009). This book is an authoritative Persian and traditional encyclopedia on medical materials (Karimi et al., 2021). In addition, symptoms of COVID-19 infection match with the Iranian traditional medicine General Ontology (Naghizadeh et al., 2021) knowledge base (http://ir-go.net). Finally, by searching its mechanism of action in articles and in combination with Persian medicine, the lavender syrup was prepared to be evaluated in this study.

The samples of cultivated *L. angustifolia* aerial parts were purchased from the local medicinal plants market, Karaj, Iran. In the next step, the plant was identified and deposited under the herbarium number (PMP-2327) by botanists at the Herbarium of Faculty of Pharmacy, Tehran University of Medical Sciences.

For preparation of syrup, aerial parts of lavender (100 g) were washed and dried at room temperature and a powder was made using a grinder. The powder was then macerated with aqueous ethanol to obtain the plant extract. The solvent was evaporated in a rotary evaporator under reduced pressure at 40°C. From the obtained dry extract, the syrup was prepared according to the USP syrup-making guideline. The prepared syrup was poured into 120 ml sterile jars, sealed, and sterilized in an autoclave. Microbial control was also performed.

**Standardization based on rosmarinic acid via HPLC**

An HPLC (high-performance liquid chromatography) method was carried out to standardize the syrup based on the content of rosmarinic acid as a main secondary metabolite in the lavender extract.

A C18 packed column (Eclipse –XBD, 25 cm ×4.6 cm ×5 µm) with a Knauer- K1001 pump was used for the separation and the column was thermostated at 20±1°C. The mobile phase was used as the gradient elution with A and B solvents (A: phosphoric acid, acetonitrile, and water (1:19:80 V/V/V); B: phosphoric acid, methanol, and acetonitrile (1:40:59 V/V/V)) at a flow rate of 1.2 ml/min as reported in Table 1. The UV/Vis detector (Knauer- UV K250) was set at 330 nm (λmax=330).

**Table 1:** Gradient profile

Time (min)	Mobile phase A (V/V) (%)	Mobile phase B (V/V) (%)
0-20	100 → 55	0 → 45
20-25	55 → 0	45 → 100

**Clinical evaluation**

**Sample size**

To achieve a power of 80%, α = 0.05 and assuming a 10% loss to follow-up, a sample size of 42 patients was calculated. The block randomization method was used to allocate the patients into two groups. All possible forms of blocks containing four interventions (A, A, B, and B) were specified and then selected randomly using the Table of randomized numbers. Finally, a random list consisting of participants’ numbers and their assigned intervention was created.

**Intervention**

In the intervention group, the participants received 9 ml of lavender syrup twice a day (18 ml daily) plus standard interventions and the control group patients received only the standard interventions based on the Iranian Ministry of Health and Medical Education's protocol (2020) such as Chloroquine phosphate/Hydroxychloroquine**,** Lopinavir/Ritonavir**, **Atazanavir/Ritonanir (Rahmanzade, 2020), for 21 days. The patients were followed up six times as follows: at the beginning of the study, during a face-to-face visit, 7, 14, 21, and finally 28 days after entering the study through telephone, respectively.

**Outcome**

The primary outcome was improvement (recovery rate) of clinical symptoms of COVID-19 after 21 days of intervention. Definition of the recovery rate of clinical symptoms was lack or the lowest frequency of COVID-19 symptoms. The secondary outcomes were patient’s satisfaction and anxiety.

These outcomes and patients’ satisfaction were evaluated by a self-assessment questionnaire based on the Visual Analogue Scale (VAS), which consisted of 15 questions and the response of each question was scored from zero to ten. In addition, anxiety levels were measured by the Hamilton questionnaire which had 14 questions and the score of each question was assessed with 0-4 points. Safety and possible side effects of the lavender syrup were evaluated at each time of visit or telephone follow-up according to Common Terminology Criteria for Adverse Events (CTCAE) version 5.0. 2017.

**Statistical methods**

The data were analyzed with SPSS software (Version 17). The normal distribution of variables was confirmed by Kolmogorov-Smirnov test. The mean±standard deviation (SD) or a number and frequency percentage were employed to describe the variables. Comparison of qualitative variables between the groups was done using the Chi-square or Fisher exact test. Quantitative variables were compared between the groups using *t*-test or Mann-Whitney U test. The time effect was assessed by the Friedman test. 

## Results

**HPLC analysis**

**Figure 1:**
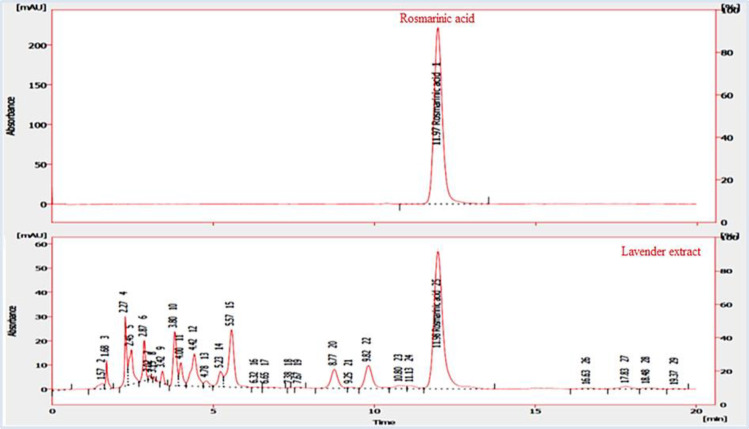
Reversed-phase HPLC analysis of the lavender extract and rosmarinic acid at 330 nm

Figure 1 shows the HPLC chromatogram of the lavender extract based on rosmarinic acid with a retention time of 11.97 min. Additionally, the rosmarinic acid amount in the syrup was 2.38 µg/mg according to the calibration curve. 

**Baseline characteristics**

**Figure 2:**
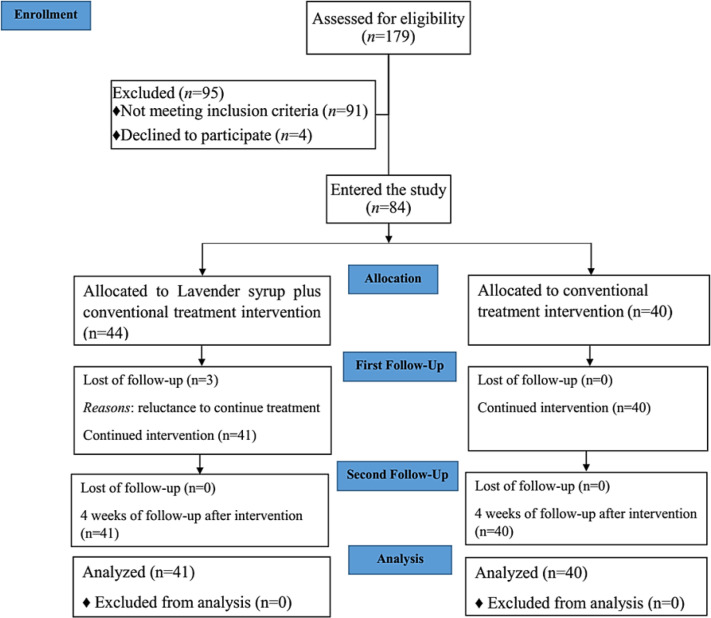
CONSORT flowchart of the study

As shown in the CONSORT flow diagram (Figure 2), between July and September, 2020; 179 participants were screened, ninety-five people were excluded from the study because 4 withdrew consent and 91 people did not meet the inclusion criteria. Finally, 84 participants were included and were randomly allocated to the lavender syrup plus conventional treatment interventions (n=44) or conventional treatment interventions only (Control group, n=40). Three participants withdrew from continuing lavender syrup before week 2 (reluctance to continue treatment, no side effects). The participants were aged 18-65 years, and 29 (70.9%) in the Lavender syrup group were male (Table 2). All participants were from east Golestan, Gonbad-E-Kavoos, Iran. The PCR test of 59 participants was positive (31 and 28 patients in lavender and control groups, respectively). Regarding the frequency of symptoms on the day of observation, fatigue and cough were the most common clinical signs, 74 (91%) participants had fatigue, whereas 66 (81%) participants had cough, followed by malaise and headache (Table 3). 

**Table 2:** Baseline characteristics of the patients with COVID-19

	Lavender syrup group	Control group	p-value
Age (years) (mean±SD)	36.7±9.75	39.07±11.03	0.309
Sex N (%)	29 (70.9) Male 12 (29.3) Female	16 (40) Male 24 (60) Female	0.007
BMI (mean±SD)	26±4.22	27.44±4.6	0.146
Onset of disease (day) Median (IQR)	7(5)	6(3)	0.126
^#^Other diseases N (%)	4 (10.3)	8 (20.5)	0.347
Temperature (℃) (mean±SD)	36.55±0.29	36.53±0.28	0.774
O_2_ saturation (%) (mean±SD)	97.92±1.42	97.87±1.36	0.867
RR (mean±SD)	17.61±0.78	17.90±0.63	0.079
PR (mean±SD)	87.87±11.3	89.37±15.1	0.616

RR: Respiratory rate (number/minute); PR: Pulse rate (beat/min); SD=standard deviation; BMI: Body mass index. IQR: interquartile range. #: Such as diabetes mellitus, cardiovascular diseases, respiratory diseases, Hypothyroidism, Hyperthyroidism, etc…

**Table 3:** The frequency of the symptoms in the study sample.

**Symptoms**	**N (%)**
**Fatigue**	74 (91)
**Cough**	66 (81)
**Malaise**	62 (76)
**Headache**	62 (76)
**Dyspnea**	46 (56)
**Anorexia**	54 (66)
**Chill**	34 (42)
**Sore throat**	52 (64)
**Chest pain**	46 (57)
**Anosmia/hyposmia**	43 (53)
**Ageusia**	39 (48)
**Diarrhea**	33 (41)
**Vomiting**	35 (43)
**Dizziness**	52 (64)
**Abdominal pain**	35 (43)

**Efficacy of the treatment**

**Primary endpoint**

All symptoms decreased in both groups with no statistically significant differences between the groups except for cough and anosmia (Table 4). 

**Figure 3:**
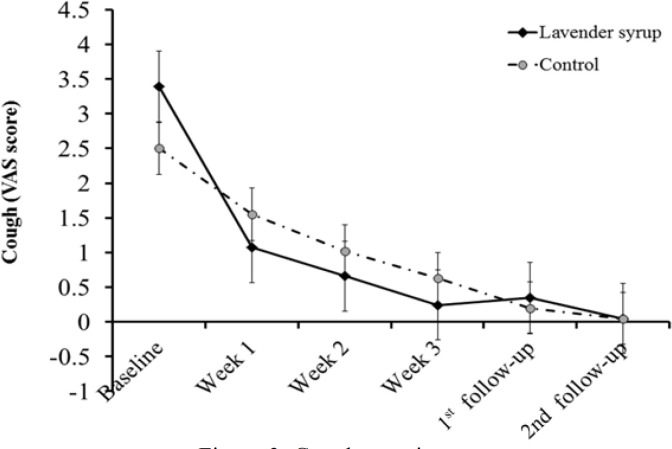
Cough severity

Of the 66 patients who had cough at baseline, cough severity was decreased in the lavender group more than the control group (p=0.02). The effect size was 0.6 for cough relief. Changes in the severity of cough were statistically higher in the lavender group than the controls at week 2 and 3 (p=0.036 and p=0.012, respectively). In addition, the 2nd and 4^th^ weeks of follow-up (week 5 and week 7 of study) after a 21-day treatment showed a reduction of the cough severity VAS score (Figure 3 and Table 4).

Of the 43 patients who had anosmia at baseline, the severity of anosmia decreased in the lavender syrup group more than the control (6.6±2.93 vs. 4.3±4.07, respectively) (p=0.043). The ageusia and other clinical symptoms in the lavender group were improved more than the control group within 3 weeks of the treatment period, but they were not statistically significant. However, larger RCTs are needed to obtain more robust results.

**Table 4:** The symptom severity of COVID-19 patients in both groups

p-value**	Changes ¥	p value*	Week 3 Mean (SD)	Week 2 Mean (SD)	Week 1 Mean (SD)	Baseline Mean (SD)	N	Group	Symptoms
**0.024**	3.63(2.3)	<0.001	0.27(0.84)	0.72(1.71)	1.13(1.86)	3.86(2.23)	36	1.00	**Cough**
	2.5(1.54)	<0.001	0.83(1.23)	1.23(2.09)	2(2.25)	3.33(1.78)	30	2.00	
**0.992**	1.67(2.76)	<0.001	0.4(1.19)	0.52(1.17)	1(1.61)	2.07(2.57)	23	1.00	**Dyspnea**
	1.69(2.33)	<0.001	0.38(1.09)	0.87(1.64)	0.92(1.56)	2.07(2.38)	23	2.00	
**0.506**	1.95(3.04)	<0.001	0.12(0.55)	0.24(0.91)	0.14(0.69)	2.07(2.9)	19	1.00	**Chill**
	1.48(2.54)	<0.001	0.05(0.32)	0.3(1.36)	0.3(1.1)	1.53(2.57)	15	2.00	
**0.828**	3.61(3.22)	<0.001	0.25(1.2)	0.38(1.18)	0.74(1.48)	3.87(3.27)	31	1.00	**Malaise**
	3.4(3.35)	<0.001	0.4(1.19)	1.17(2.59)	1.42(2.24)	3.85(3.45)	31	2.00	
**0.246**	5.15(2.88)	<0.001	0.48(1.33)	0.87(1.65)	1.92(2.48)	5.62(3.04)	39	1.00	**Fatigue**
	4.37(3.54)	<0.001	0.67(1.73)	1.42(2.33)	2.57(2.86)	5.05(3.5)	35	2.00	
**0.635**	1.32(2.09)	<0.001	0(0)	0.35(1.54)	0.47(1.3)	1.32(2.09)	17	1.00	**Diarrhea**
	1.2(2.51)	<0.001	0.17(0.72)	0.17(1.12)	0.66(2.18)	1.37(2.3)	16	2.00	
**0.369**	1.87(2.55)	<0.001	0(0)	0.35(1.38)	0.56(1.41)	1.87(2.55)	20	1.00	**Vomiting**
	1.61(2.86)	<0.001	0(0)	0.25(1.19)	0.62(1.65)	1.6(2.86)	15	2.00	
**0.633**	2.95(3.08)	<0.001	0.17(0.76)	0.8(1.82)	1.07(2.06)	3.12(3.02)	25	1.00	**Dizziness**
	2.57(3.22)	<0.001	0.45(1.29)	0.75(1.85)	1.02(2.31)	3.02(3.26)	27	2.00	
**0.697**	1.5(2.72)	<0.001	0.17(0.78)	0.17(0.84)	0.37(0.89)	1.67(2.52)	17	1.00	**Abdominal pain**
	1.84(2.88)	<0.001	0.26(1.15)	0.15(0.67)	1(2.04)	2.1(2.86)	18	2.00	
**0.635**	3.5(3.28)	<0.001	0.35(1.12)	0.9(2.09)	1.07(2.08)	3.85(3.25)	32	1.00	**Headache**
	3.89(3.31)	<0.001	0.17(0.68)	0.69(1.79)	1.87(3.06)	4.07(3.49)	30	2.00	
**0.132**	3.36(3.17)	<0.001	0.09(0.43)	0.12(0.55)	0.75(1.28)	3.46(3.24)	28	1.00	**Sore throat**
	2.32(3.01)	<0.001	0.15(0.57)	0.37(1.33)	0.57(1.72)	2.47(2.94)	24	2.00	
**0.144**	3.35(3.23)	<0.001	0.15(0.8)	0.7(1.78)	1.55(2.48)	3.5(3.19)	27	1.00	**Anorexia**
	3.05(3.42)	<0.001	0.2(0.86)	0.84(2.19)	1.38(2.4)	3.25(3.41)	27	2.00	
**0.653**	1.9(2.65)	<0.001	0.46(1.46)	0.35(1.01)	0.64(1.4)	2.41(2.79)	22	1.00	**Chest pain**
	2.22(3.04)	<0.001	0.42(1.15)	0.9(2.38)	1.15(2.16)	2.65(3.12)	24	2.00	
**0.036**	6.6(2.93)	<0.001	0.26(0.86)	2.26(3.55)	3.43(3.67)	6.86(3.04)	23	1.00	**Anosmia**
	4.3(4.07)	<0.001	1(3.24)	1.65(3.24)	2.21(2.43)	5.3(3.45)	20	2.00	
**0.082**	5.5(3.5)	<0.001	0(0)	2.23(3.53)	3.23(3.36)	6.04(3.18)	21	1.00	**Ageusia**
	3.5(4.07)	<0.001	0.61(1.91)	0.83(2.17)	1.77(2.34)	4.5(3.41)	18	2.00	

Group 1=Lavender syrup and group 2= control; *: Friedman test; **: Changes from baseline to week 3; ***: Mann-Whitney U test

**Secondary outcomes**

The Hamilton total score was decreased in both groups after 3 weeks and there was no significant difference between the groups at baseline and the end of the treatment period (Table 5).

Finally, patients’ satisfaction score in the Lavender group (7.74±2.42) was greater than the control (6.87±2.19) at the end of the observation period (p=0.043). Two and four weeks after the intervention (follow-up period), there was no significant difference between the groups in terms of severity of symptoms.

**Safety of the treatment**

There were no complications or adverse events related to lavender syrup during the study.

**Table 5:** Total Hamilton questionnaire scores before and after 3 weeks of the intervention

	Group	Mean	SD	p-value
Sum Hamilton-before	1	13.8378	8.732170	0.57
	2	15.5405	10.13409	
Sum Hamilton-after	1	3.10000	6.792910	0.35
	2	2.65790	5.189780	

SD: standard deviation. Group 1=Lavender syrup and group 2= control

## Discussion

This single-center open-labeled, randomized, controlled clinical trial describes the efficacy of lavender syrup against the cough of patients with COVID-19. All clinical features of participants with COVID-19 were diminished in both the control and lavender groups. Furthermore, the lavender syrup caused a robust reduction in cough compared with the control group during the 21-day treatment. Also, the improvement of anosmia was higher in the lavender group than in the control group.

COVID-19 is associated with symptoms such as fever, severe pneumonia, olfactory and gustatory dysfunction, and acute cardiac injury (Delgado-Roche and Mesta, 2020; Hajikhani et al., 2020). After entry into the host cells, COVID-19 may cause damage through inflammation and reactive oxygen species (ROS) production. Airway inﬂammation causes oxidative stress and ROS, which are noxious stimuli and threaten airway function (Taylor–Clark, 2015). Therefore, oxidative stress may activate sensory nerves to trigger cough. Notably, the lung is easily affected by exogenous ROS because of its anatomy, function, and location, which can result in lung diseases (Chamitava et al., 2020). It has been demonstrated that high levels of cytokines and chemokines in COVID-19 patients, indicate a cytokine storm leading to severe tissue damage and organ failure (Huang et al., 2020). Viral infections in the airways induce ROS formation that causes cellular damage and ensuing inflammation (Komaravelli and Casola, 2014). The crosstalk between inflammation and oxidative stress has been established (Sies, 2015).

Moreover, phytochemical analysis of the *Lavander* species showed that they have many potential secondary metabolites, such as phenolic acids, flavonoids, coumarins, diterpenes, triterpenes, and tannins. Likewise, it has been reported that lavender is an effective herbal medicine in treating headache, inflammation, stress, and oxidative stress. Thus, the lavender extracts display their antioxidant and anti-inflammatory activities due to their rich content of phenolic compounds such as rosmarinic acid (Kashani et al., 2011; Hawrył et al., 2019).

In a previous report from this trial, we reported the beneficial effect of lavender syrup (n=23) on olfactory dysfunction. Besides, we also mentioned the safety of the lavender syrup as it caused no side effects (Hashem-Dabaghian et al., 2022).

A previous animal study on asthma indicated that oral administration of *Lavandula dentata* extract (300 mg/kg) in guinea pigs showed a significant reduction of IgE, triglycerides, total cholesterol, glucose levels in serum and exerted antioxidant activity by reducing ROS, malondialdehyde (MDA) and increasing glutathione (GSH) levels in lungs after the 21-day treatment. They showed that lavender suppressed asthma and airway inﬂammation as well as alteration of antioxidant defense through its antioxidant activity (Almohawes and Alruhaimi, 2019).

It is well known that rosmarinic acid has anti-inflammatory, anti-allergenic, anti-viral, antimicrobial, cardioprotective, immunomodulatory and antioxidant effects (Liang et al., 2020; Costa et al., 2012; Liang et al., 2016). Likewise, rosmarinic acid significantly blunted inflammation/oxidation stress-induced lung injury via upregulation Cu/Zn superoxide anion dismutase (SOD), plasmatic glutathione peroxidase (GPx), and catalase (CAT) activities along with down-regulation of NADPH oxidase 2 and 4 **(**NOX-2 and NOX-4) expression, which led to the suppression of T helper 1/2 (Th1/Th2) cytokine, interferon gamma (IFN-γ), interleukin 4 (IL-4), IL-5, and IL-13 in lung tissues (Liang et al., 2020). Costa et al. showed the immunomodulatory potential of rosmarinic acid which was mediated by reducing the number of leukocytes/eosinophils, eosinophil peroxidase activity, respiratory tract, and IL-4 in a murine model of respiratory allergy (Costa et al., 2012). Additionally, rosmarinic acid exerted anti-inflammatory and anti-asthmatic properties by inhibiting inflammatory cytokines including nuclear factor-κB (NF-κB) and ERK/JNK/p38 as mitogen-activated protein kinase (MAPK) family and acidic mammalian chitinase (AMCase), CCL11(eotaxin), CCR3, Ym2 and E-selectin in a murine model of asthma (Liang et al., 2016).

Moreover, it is known that hypertension and cardiac dysfunction are the most important complications in patients with COVID-19. These comorbidities occur via using angiotensin-converting enzyme 2 receptor (ACE-2) and transmembrane Serine Protease 2 (TMPRSS-2) receptors by SARS-CoV-2 for cellular entry (Nasiri et al., 2020). Thus, one of the reasons for these comorbidities is low levels of ACE-2 receptors along with an increase in renal potassium excretion and sodium retention, which lead to hypokalemia and hypernatremia (Beck, 2020). Furthermore, inhibition of ACE caused an increase in bradykinin levels and bronchoconstriction, which induced cough, a common symptom seen in COVID-19 patients (Al-Shamlan and El-Hashim, 2019; Mahmoudpour et al., 2013). Interestingly, in this study, lavender improved cough, and olfactory and gustatory dysfunction compared with the control group, and hence we believe lavender may be effective (directly/indirectly) in COVID-19 cardiac dysfunction via suppressing cough and regulating the interplay between ACE-2 and bradykinin. 

Moreover, the results of our study suggest the potential of lavender as a complementary therapy against most of clinical symptoms of COVID-19 and may be administered to hospitalized patients to achieve more efficient.

This study had the following limitations: Due to open-label design of the study (owing to pandemic conditions) and the small sample size, the power of the present study might be lower than that to detect any differences between the groups for the secondary outcome variables. Another major limitation of our study was that we could not perform RT-PCR testing of all enrolled participants due to the prevailing situation. Moreover, laboratory tests were not obtained for patients, and thus we could not determine antioxidant and inflammatory changes before and after the observation. 

The findings of the present study may highlight the efficacy of lavender syrup as an adjunct to standard care in providing the symptomatic improvement of COVID-19. The severity of cough in these patients was significantly ameliorated by lavender syrup. However, considering the limitations of this study, we emphasize the preliminary nature of the present findings, which necessitates further clinical trials with larger population size and considering solid outcomes related to COVID-19 mortality. Such data is required to generate more reliable evidence of the efficacy of lavender syrup in improving the severity of COVID-19.

## Conflicts of interest

The authors have declared that there is no conflict of interest.
